# Supplementary material for: Preclinical Evaluation of Nanoemulsion and Polymeric Nanocapsule Delivery Systems of 4-(Phenylselanyl)-2H-Chromen-2-One for Rheumatoid Arthritis and Comorbidities
Source: Pharmaceuticals (Basel). 2025 Sep 16;18(9):1379. doi: 10.3390/ph18091379 (PMC12472991; doi:10.3390/ph18091379)
Supplement: Supplementary file 1 [file pharmaceuticals-18-01379-s001.zip › pharmaceuticals-3820745-supplementary.pdf]

SUPPLEMENTARY FIGURES

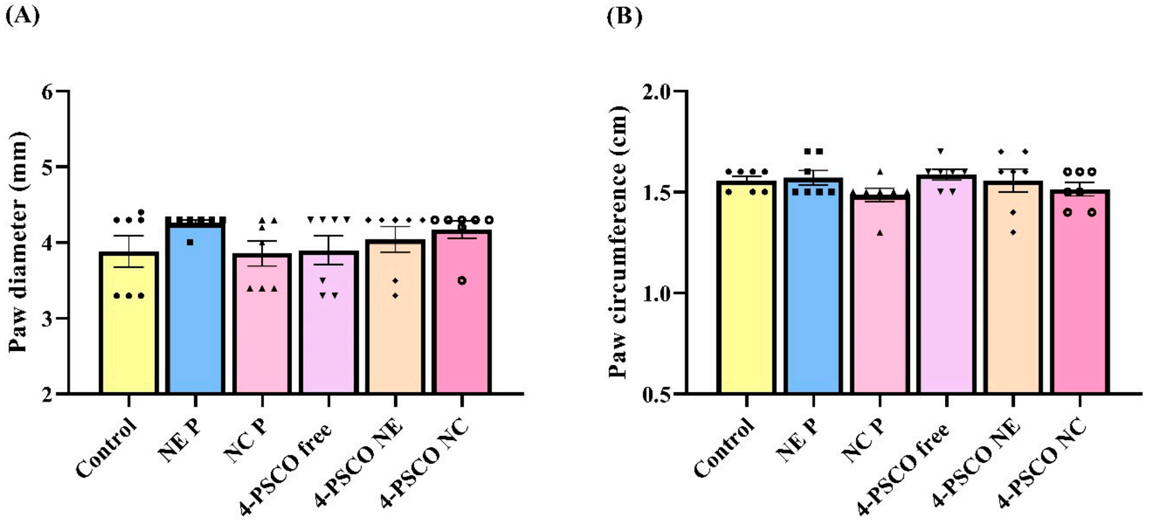

Figure S1. Effects of free 4-(phenylselanyl)-2H-chromen-2-one (4-PSCO), 4-PSCO-loaded nanoemulsions (4-PSCO NE), and 4-PSCO-loaded nanocapsules (4-PSCO NC) (1 mg kg-1, i.g.) on paw diameter **(A)** and circumference **(B)** induced by Complete Freund's Adjuvant (CFA) (0.1 mL, i.pl.) in mice. Each point represents the mean of 7 female mice in each group (one-way ANOVA followed by Tukey's test) ($p > 0.05$).

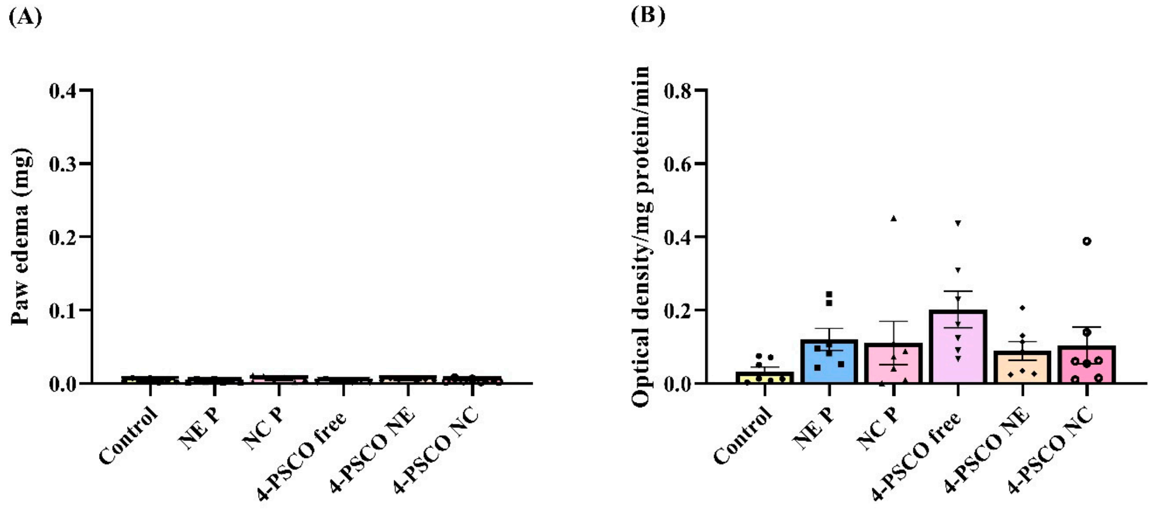

Figure S2. Effects of free 4-(phenylselanyl)-2H-chromen-2-one (4-PSCO), 4-PSCO-loaded nanoemulsions (4-PSCO NE), and 4-PSCO-loaded nanocapsules (4-PSCO NC) (1 mg kg-1, i.g.) on paw edema **(A)** and myeloperoxidase (MPO) activity **(B)** induced by Complete Freund's Adjuvant (CFA) (0.1 mL, i.pl.) in mice. Each point shows the mean of 7 female mice per group (one-way ANOVA followed by Tukey's test) ($p > 0.05$).

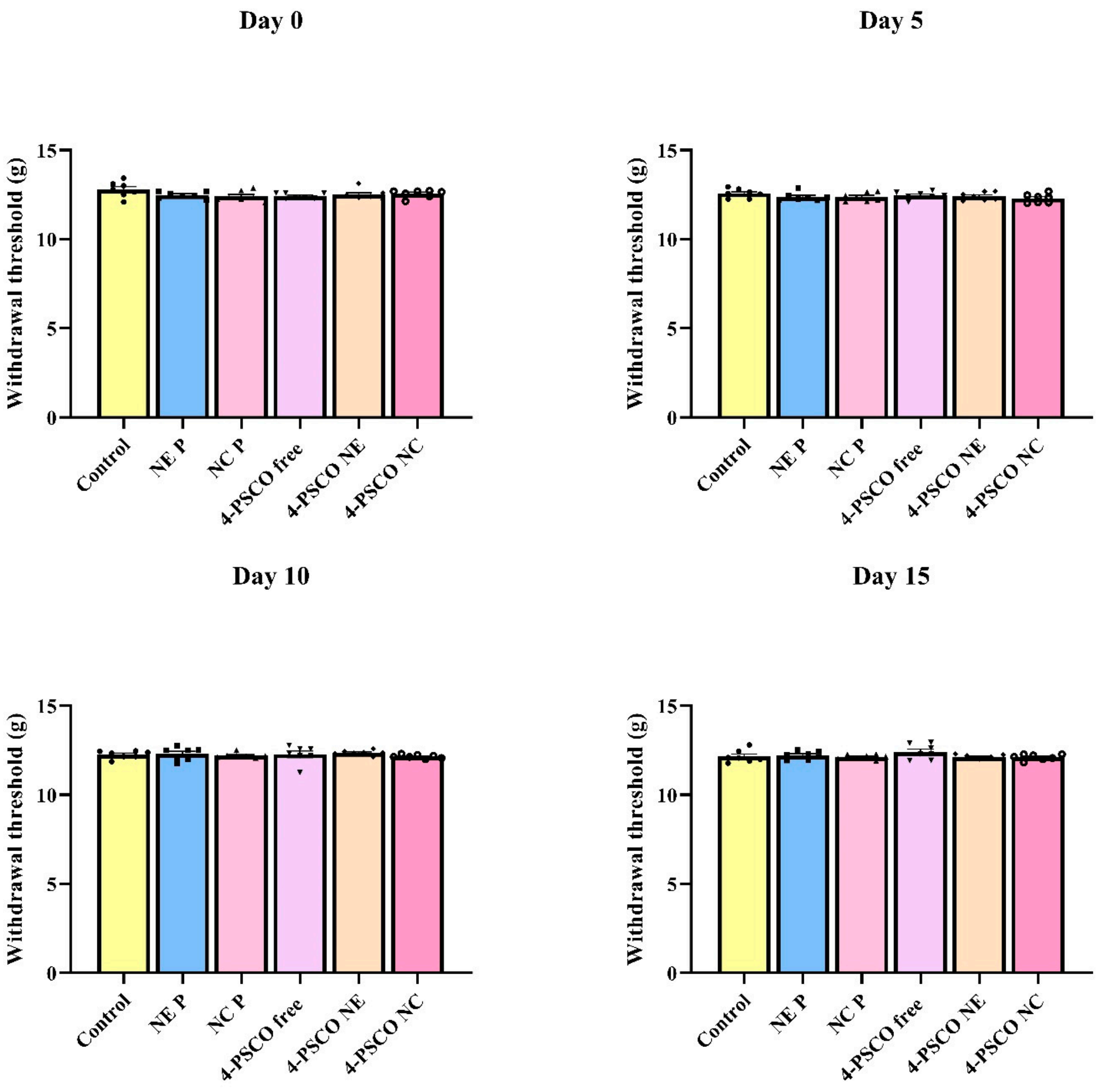

Figure S3. Effects of free 4-(phenylselanyl)-2H-chromen-2-one (4-PSCO), 4-PSCO-loaded nanoemulsions (4-PSCO NE), and 4-PSCO-loaded nanocapsules (4-PSCO NC) (1 mg kg^{-1}, i.g.) on paw withdrawal threshold for mechanical stimuli in the von Frey test induced by Complete Freund's Adjuvant (CFA) (0.1 mL, i.pl.) in mice. Each point represents the mean of 7 female mice in each group (one-way ANOVA followed by Tukey's test) ($p > 0.05$).

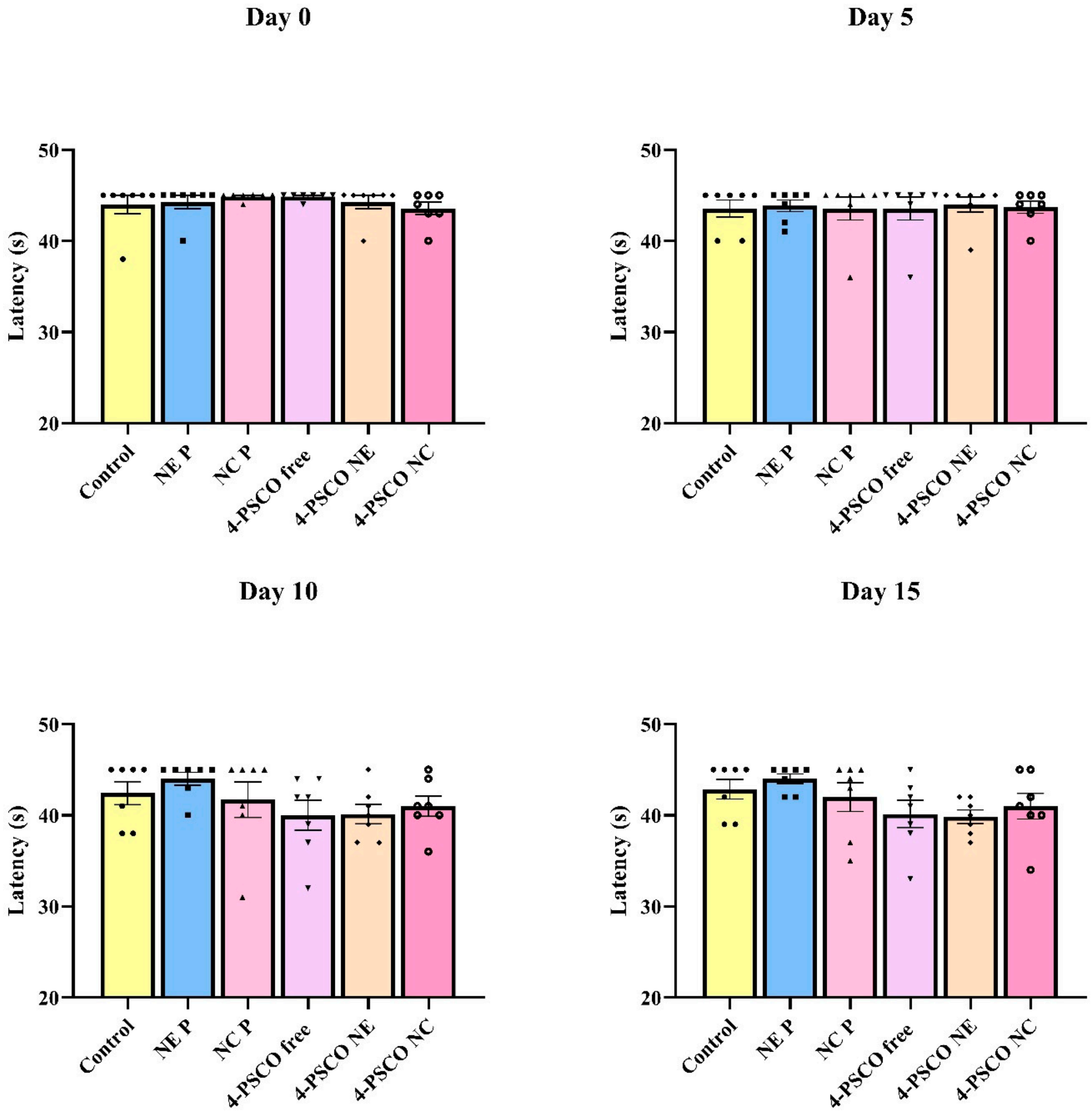

Figure S4. Effects of free 4-(phenylselanyl)-2H-chromen-2-one (4-PSCO), 4-PSCO-loaded nanoemulsions (4-PSCO NE), and 4-PSCO-loaded nanocapsules (4-PSCO NC) (1 mg kg-1, i.g.) on thermal hyperalgesia in the hot plate test induced by Complete Freund's Adjuvant (CFA) (0.1 mL, i.pl.) in mice. Each point represents the mean of 7 female mice in each group (one-way ANOVA followed by Tukey's test) ($p > 0.05$).

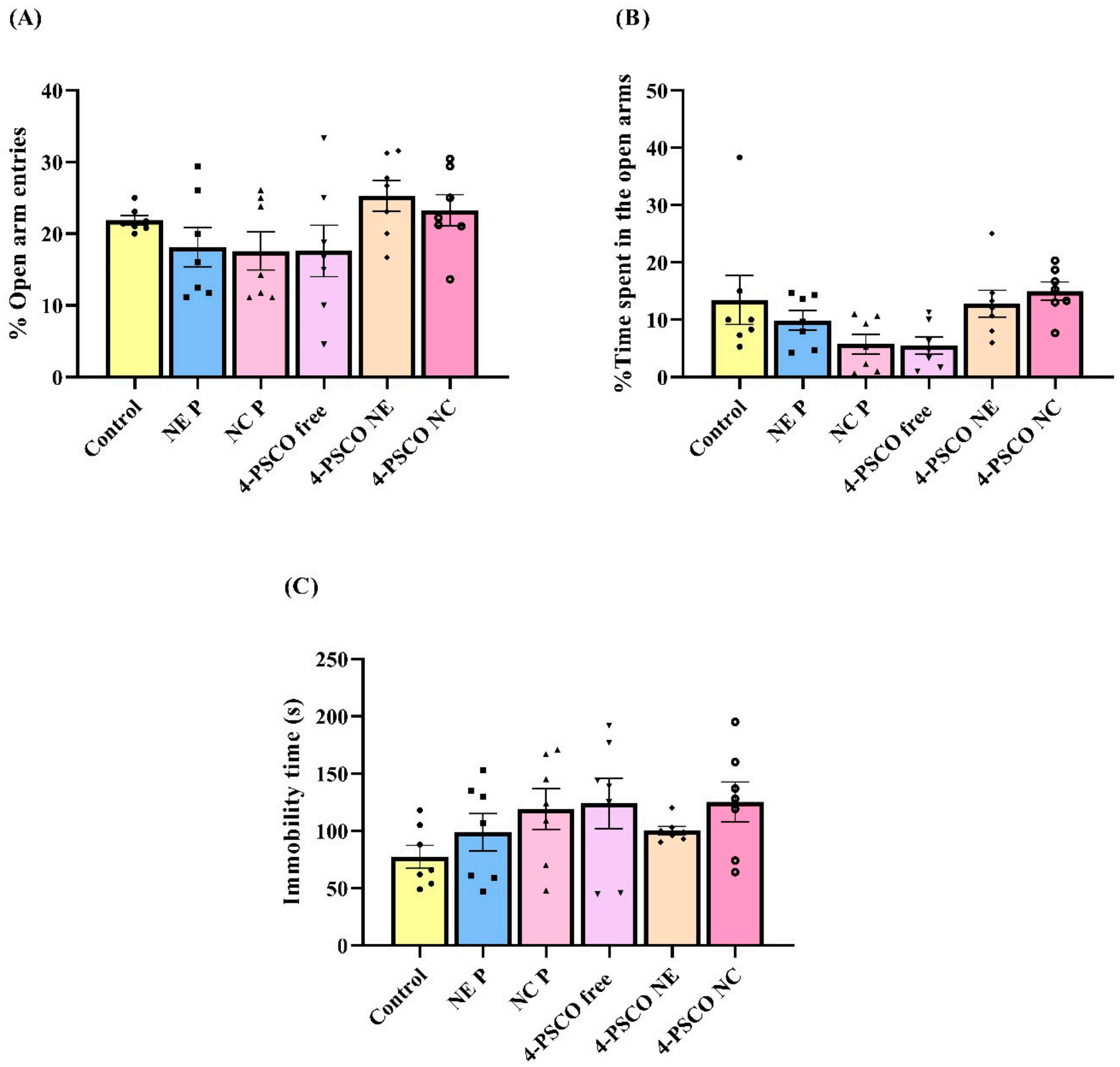

Figure S5. Effects of free 4-(phenylselanyl)-2H-chromen-2-one (4-PSCO), 4-PSCO-loaded nanoemulsions (4-PSCO NE), and 4-PSCO-loaded nanocapsules (4-PSCO NC) (1 mg kg^{-1}, i.g.), and Complete Freund's Adjuvant (CFA) (0.1 mL, i.pl.) on the percentage of entries into the open arms **(A)**, percentage of time spent in the open arms **(B)**, and immobility time **(C)** induced by CFA (0.1 mL, i.pl.) in mice. Each point represents the mean of 7 female mice per group (one-way ANOVA followed by Tukey's test) ($p > 0.05$).

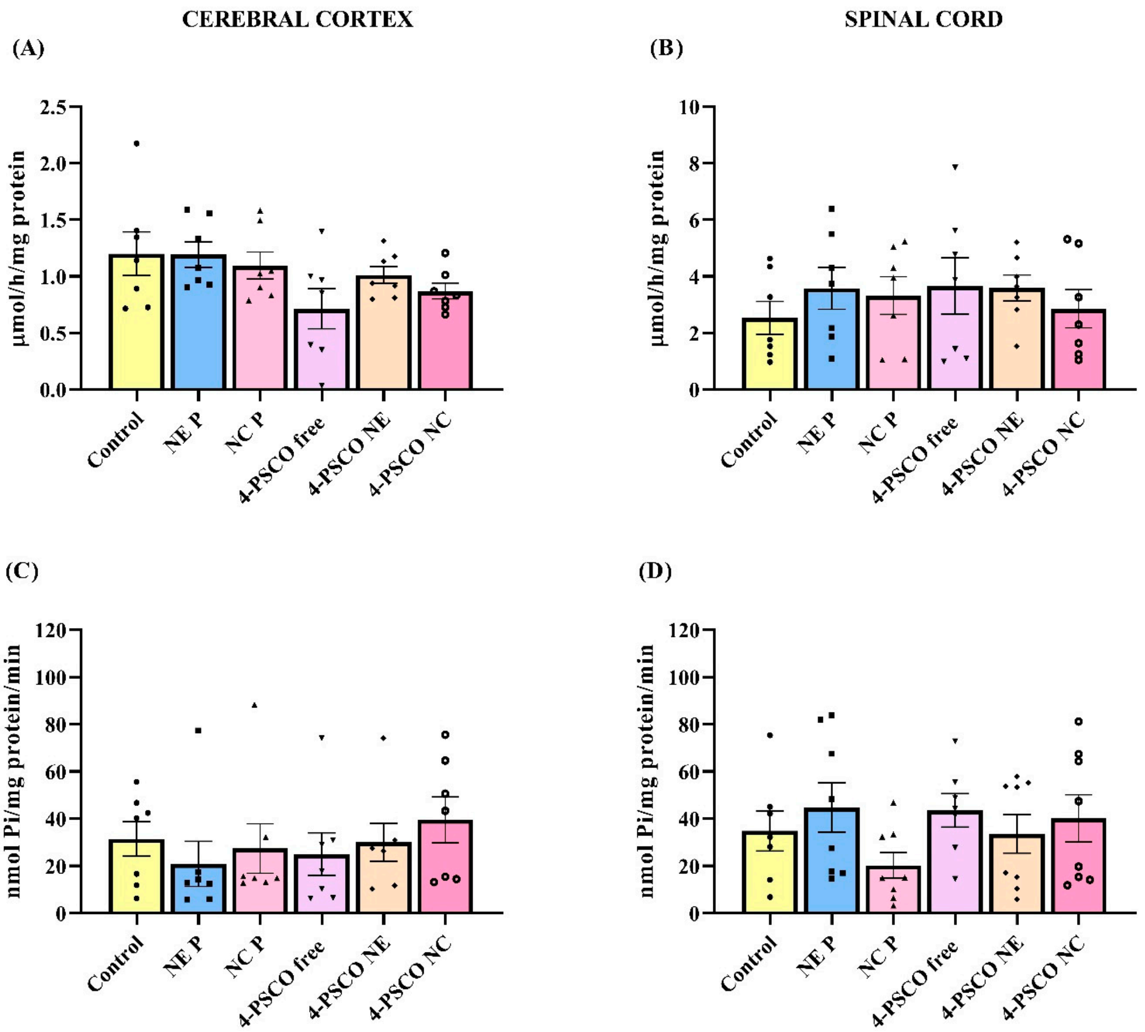

Figure S6. Effects of free 4-(phenylselanyl)-2H-chromen-2-one (4-PSCO), 4-PSCO-loaded nanoemulsions (4-PSCO NE), and 4-PSCO-loaded nanocapsules (4-PSCO NC) (1 mg kg-1, i.g.) on AChE activity in the cerebral cortex **(A)** and spinal cord **(B)**; and $Na^{+}K^{+}$-ATPase activity in the cerebral cortex **(C)** and spinal cord **(D)** induced by Complete Freund's Adjuvant (CFA) (0.1 mL, i.pl.) in mice. Each point represents the mean of 7 female mice per group (one-way ANOVA followed by Tukey's test) ($p > 0.05$).

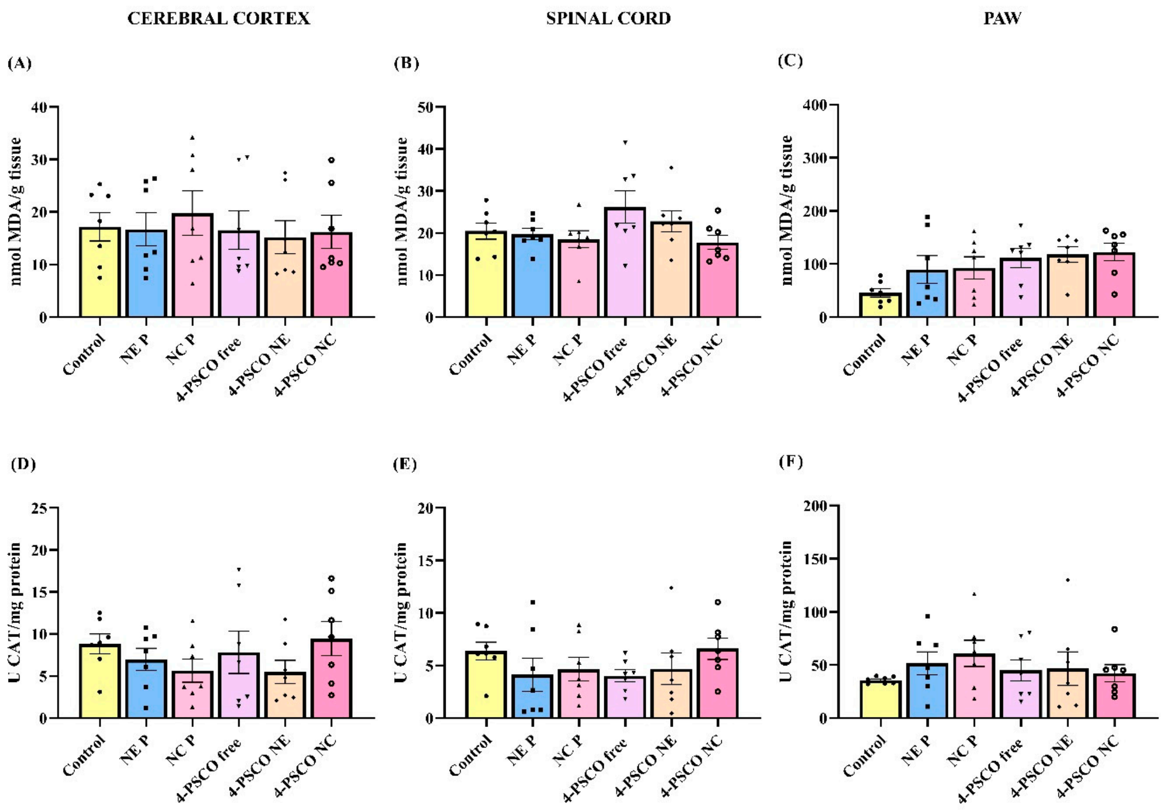

Figure S7. Effects of free 4-(phenylselanyl)-2H-chromen-2-one (4-PSCO), 4-PSCO-loaded nanoemulsions (4-PSCO NE), and 4-PSCO-loaded nanocapsules (4-PSCO NC) (1 mg kg-1, i.g.) on TBARS levels in the cerebral cortex **(A)**, spinal cord **(B)**, and paw **(C)**; CAT activity in the cerebral cortex **(D)**, spinal cord **(E)**, and paw **(F)** induced by Complete Freund's Adjuvant (CFA) (0.1 mL, i.pl.) in mice. Each point represents the mean of 7 female mice per group (one-way ANOVA followed by Tukey's test) ($p > 0.05$).
